# Exploring the Role of Dynapenic Obesity Phenotype in Mental Health among Korean Older Adults: A Screening Perspective

**DOI:** 10.1002/alz70860_100687

**Published:** 2025-12-23

**Authors:** Jeonghyeon Kim, Taewan kim, Yoonhwan Kim, Jiho Shin, Jinkyung Cho, Donghyun kim, Youngyun Jin

**Affiliations:** ^1^ Sungkyunkwan University, suwon, GYEONGGI‐DO, Korea, Republic of (South); ^2^ Hanbat National University, Daejeon, Korea, Republic of (South)

## Abstract

**Background:**

Dynapenic obesity, characterized by reduced muscle strength and increased adiposity, is associated with elevated risks of depression and cognitive decline. This study investigates dynapenic obesity phenotypes as a predictor of depressive symptoms and cognitive impairment and evaluates its mediating role between these mental health outcomes.

**Method:**

We included 938 community‐dwelling Korean older adults aged 65–90 years (72.9% women, mean age 73.4 ± 5.6) without a history of depression or dementia. Dynapenia was defined by handgrip strength (HGS) <26 kg for men and <18 kg for women, based on Asian Working Group for Sarcopenia (AWGS) criteria. General obesity was classified by body mass index (BMI), with a normal range (18.5–24.9 kg/m^2^) and obesity (≥25.0 kg/m^2^) categories, while abdominal obesity was defined as waist circumference (WC) ≥90 cm for men and ≥85 cm for women. Dynapenic obesity (DO) was defined as low HGS with obesity, and dynapenic abdominal obesity (DAO) as low HGS with abdominal obesity. Cognitive function was assessed using the Mini‐Mental State Examination for Dementia Screening (MMSE‐DS), and depressive symptoms were measured using the Center for Epidemiological Studies Depression Scale (CES‐D). Multivariable logistic regression and mediation analyses were performed.

**Result:**

Multivariable logistic regression showed that participants with dynapenia (OR=2.39, 95% CI=1.368‒4.157, *p* = 0.002), DO (OR=2.20, 95% CI=1.18‒3.93, *p* = 0.012), DAO (OR=3.20, 95% CI=1.923‒5.253, *p* <0.001), and depressive symptoms (OR=3.10, 95% CI=1.911‒5.037, *p* = 0.002) had a higher risk of cognitive impairment. Similarly, those living alone (OR=3.81, 95% CI=2.267‒6.415, *p* <0.001), with dynapenia (OR=3.11, 95% CI=1.879‒5.158, *p* <0.001), DO (OR=2.04, 95% CI=1.056‒3.948, *p* = 0.034), and cognitive impairment (OR=3.34, 95% CI=2.058‒5.422, *p* <0.001) were at a higher risk of depressive symptoms. Additionally, mediation analysis revealed that DO accounted for 22.2% of the indirect effect between depressive symptoms and cognitive function and 24.1% between cognitive function and depressive symptoms, while no significant indirect effect was observed for DAO.

**Conclusion:**

Dynapenic obesity, defined by handgrip strength and BMI, significantly contributes to cognitive impairment and depressive symptoms in Korean older adults. These findings underscore the importance of considering dynapenic obesity in mental health screenings and interventions.

